# Ex-Press Mini-Implant in the Management of Ocular Hypertension Secondary to Silicone Oil Tamponed

**Published:** 2016

**Authors:** Nicola CARDASCIA, Francesco CANTATORE, Paolo FERRERI, Luigi SBORGIA, Giovanni ALESSIO

**Affiliations:** 1 Department of Ophthalmology and Neuroscience, Policlinico di Bari, A. Moro Bari University, Italy

**Keywords:** Ex-Press Mini-Implant, Ocular Hypertension, Silicone Oil Tamponed

## Abstract

This study was designed to compare the success of patients with ocular hypertension, secondary to pars plana vitrectomy and silicone oil tamponade, who received an Ex-PRESS Glaucoma Filtration Device P50 (Alcon Laboratories, Inc. Fort Worth, Texas, USA) to those who had conventional trabeculectomy. The records of 10 eyes of 10 consecutive subjects who had Ex-press implants and 9 eyes of 9 consecutive controls who had trabeculectomy procedures were reviewed. Success was defined as the reduction of intraocular pressure (IOP) in patients who did not require further glaucoma surgery in the eye of note during the entire follow-up. IOP was reduced by 10.3 ± 9.7 mmHg (range -31 to 3) in the Ex-PRESS group and by 13.9 ± 11.4 mmHg (range -35 to -4) in the trabeculectomy group. The difference in the percentage of IOP reduction between the standard trabeculectomy group (42.7%) and the Ex-PRESS group (35.9%) was not statistically significant (P = 0.72). The Ex-PRESS device seems to be at least as effective as the standard trabeculectomy in lowering the IOP of patients with hypertension secondary to pars plana vitrectomy and silicone oil tamponade. Even though the data suggested that the Ex-PRESS device did not result in an overall greater reduction in IOP than trabeculectomy, this does not reach statistical significance.

## INTRODUCTION

Silicone oil tamponade represents an important procedure in vitreoretinal surgery, especially in cases of complicated retinal detachments (1). Intraocular silicone oil is associated with several complications (keratopathy, cataract, glaucoma, subretinal migration of silicone oil droplet, proliferation of retinal fibrous membranes) if left in the vitreous cavity for an extended period of time (2, 3). Increased intraocular pressure (IOP) is a rather common complication in eyes that have undergone pars plana vitrectomy with a silicone oil tamponade. Numerous studies have reported several risk factors for an increased IOP after silicone oil injections (4-10). Even if the uncontrolled IOP, which was induced by silicone oil tamponade, was not associated with a history of glaucoma (11), eyes with pre-existing uncontrolled ocular pressure were more likely to have postoperative pressure complications (12). It could be related to an acute pupillary block, choroidal effusions with anterior displacement of the lens–iris complex, anterior synechiae, and the migration of the emulsified oil in the anterior chamber (4-10), Glaucoma treatments are directed to balance IOP (13, 14), either pharmacologically or surgically. Surgery is suggested when pharmacological strategies fail to control IOP (15). Trabeculectomy is a conventional surgical approach (16). An alternative method could be to implant the Ex-PRESS® P50 glaucoma filtration device (Alcon Inc., Fort Worth, TX). This is a metallic, non-valved flow-restricting device, designed to reduce IOP in glaucomatous disorders (17). It was proposed as a mini-invasive surgical filtering device (18, 19), inserted under a costumed scleral flap to shunt aqueous humour from the anterior chamber to the filtration bleb (20). During the surgical filtering procedures in vitrectomized eyes, the Ex-press device could offer better compliance than regular trabeculectomy, preventing unpredictable volume changes of the anterior and posterior chambers. In this study we compared the success of patients with ocular hypertension, secondary to silicone oil tamponade, who had an Ex-PRESS® P50 mini glaucoma shunt device implantation to those who had conventional trabeculectomy. 

## MATERIALS AND METHODS

Statement of Ethics: We certify that all applicable institutional and governmental regulations concerning the ethical use of human data were followed during this research. The analysis involves data from a retrospective cohort study. Patients were recruited at a single center (Policlinico di Bari, A. Moro University of Bari, Ophthalmology Department, Italy) between January 2007 and November 2012. All patients underwent 23G pars plana vitrectomy and 1000 centistokes silicone oil tamponade. None of them were affected by glaucoma previous of retinal surgery. One surgeon (FC) performed all the surgeries for glaucoma. All surgeries were performed under local anesthesia (5 mL solution of 2.5 mL of 2% lidocaine **[**xylocaine] and 2.5 mL of 0.5% bupivicaine [Marcaine]). Eyes treated with Ex-PRESS were implanted with the Ex-PRESS® P50 device under a scleral flap. The surgical procedures were similar in both treatment arms. A limbus-based conjunctival flap was dissected, followed by a “4 × 4 mm × half the scleral thickness” scleral flap dissected up to the cornea. A cellulose microsponge soaked in 0.4 mg/mL Mitomycin-C solution was applied to the scleral flap, with the conjunctive draped over the sponge for 3 minutes. The sponge was then removed and the area was washed with irrigating saline solution. For the eyes treated with EX-PRESS® P50, the mini-implant was inserted parallel to the iris, through the “gray line” in the clear cornea. In the eyes treated with trabeculectomy, a sclerotomy associated with a peripheral iridectomy was performed. For both procedures the scleral flap was then sutured using two 10-0 nylon sutures at the edge of the flap. The conjunctiva was sutured over the limbus with one uninterrupted, single-running Vicryl suture. During the six postoperative weeks, topical corticosteroids and antibiotics were administered four times a day, along with 1% atropine, twice daily. All patients were aged 18 years or older and all presented with ocular hypertension secondary to silicone oil tamponade, not controlled by medical therapy. Patients with myopia greater than -6 diopters (D) or previous ocular filtrating glaucoma surgery were excluded. Optical assessments during the follow-up included applanation tonometry (21), to measure IOP and the Early Treatment Diabetic Retinopathy Study chart for visual acuity (VA) (22). Responses were classified as: intraocular pressure values less than 21 mmHg thresholds; no subsequent intraocular pressure medication prescribed; and no further surgery performed for ocular hypertension. Statistical analysis (analysis of variance, P < 0.5) was performed using InStat (GraphPad Software Inc, La Jolla, CA, USA).

## RESULTS

The Ex-press group was comprised of 10 eyes of 10 patients (8 men) with a mean age of 52.5 ± 13 years. Only three were affect by myopia (mean spherical equivalent: -2.75 ± 1.27 D). Lens phacoemulsification and intraocular lens implant were associated with 23G pars plana vitrectomy in 7 eyes. Ocular hypertension developed 9.2 ± 14.4 months (range = 1-49.5) after vitreoretinal surgery, EX-PRESS® P50 implant was performed 12.1 ± 14.8 months (range = 0.7-38.8) later. The pre-operative visual acuity and IOP were 1 ± 0.8 logMAR (range 0-2) and 26.2 ± 7.4 mmHg (range = 18-45). After 18.8 ± 14.3 months (range=5.5-43.8), visual acuity and IOP were 0.83 ± 0.7 logMAR (range 0-2) and 15.9 ± 5.8 mmHg (range 12-28), respectively. IOP was reduced by 35.9% (10.3 ± 9.7 mmHg; range -31 to 3).

**Figure 1:**
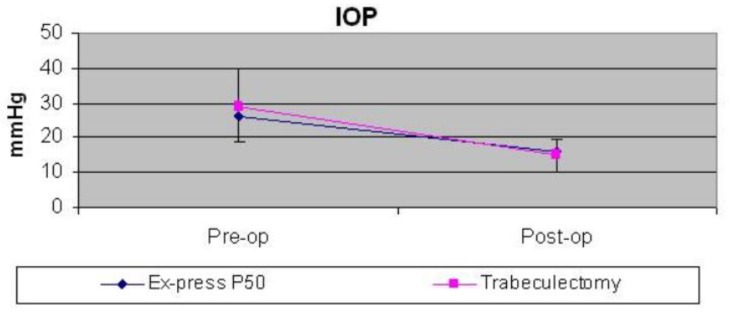
After approximately one year, IOP was reduced in the EX-Press and trabeculectomy groups, without any significant differences between them (P = 0.72)

**Figure 2:**
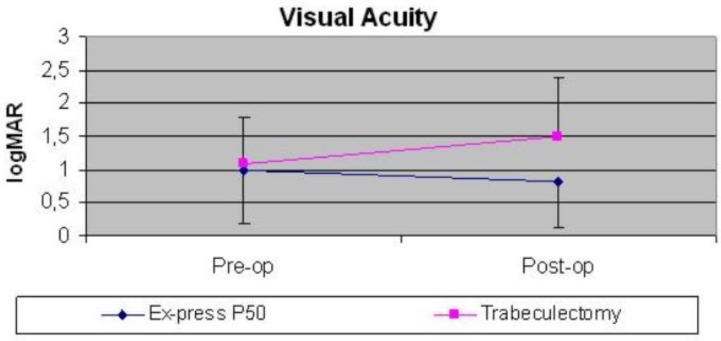
After one year of follow-up, visual acuity worsened, but not significantly (P = 0.08) in the trabeculectomy group

The trabeculectomy group was comprised of 9 eyes of 9 patients (6 women) with a mean age of 54.3 ± 16.8 years. Only four were myopic (mean spherical equivalent: -3.25 ± 1.34 D). Lens phacoemulsification and IOL implant were associated with 23G pars plana vitrectomy in 7 eyes. Ocular hypertension developed 44.9 ± 86.5 months (range 2-252.7) after retinal surgery. Medical hypotensive therapy was unsatisfactory after 5.6 ± 9.5 months (range 0.03-30.2) and those eyes were scheduled for filtrating surgery. Pre-operative visual acuity and IOP were 1.1 ± 0.7 logMAR (range 0.4-2) and 28.9 ± 11.1 (range 14-50). After 11.1 ± 10.8 months (range 3.9-39.3) visual acuity and IOP were 1.5 ± 0.9 logMAR (range 0.8-3) and 15 ± 4.7 (range 10-18), respectively. IOP was reduced by 42.7% (10 13.9 ± 11.4; range -35 to -4). In both groups, subsequent intraocular pressure medication was prescribed and further surgery was performed for ocular hypertension. Pre-operative IOP and visual acuity were similar in both groups (IOP: P = 0.54, VA: P = 0.27). After approximately one year, IOP reduced by 35.9% in the EX-Press group and by 42.7% in the trabeculectomy group (Figure 1). Visual acuity was preserved in both groups (Ex-Press: P = 0.34, Trabeculectomy: P = 0.3) (Figure 2). Any significant differences were registered between the groups (IOP: P = 0.72, VA: P = 0.08).

## DISCUSSION

Many clinical and experimental studies have emphasized the role of silicone oil in the development of high intraocular pressure in vitrectomized eyes. Mechanisms include acute pupillary block, choroidal effusions with anterior displacement of the lens–iris complex, anterior synechiae, and emulsified silicone oil droplet in the anterior chamber (4-10, 23). Electron microscopy showed that the emulsified silicone oil leaves the anterior chamber through the trabecular and uveoscleral routes (24). In eyes implanted with Ex-PRESS® P50, slit lamp biomicroscopy of the anterior segment did not reveal any silicone oil droplets under the surgical bleb. We believe the lumen of the device does not allow any silicone flow from anterior chamber to the conjunctival bleb. A study conducted on silicone oil emulsification demonstrated that the size of an oil droplet in the human anterior chamber was 0.038 ± 0.018 mm (25), which was considerably larger than the 50µ lumen of the device. Those assumptions suggest that the Ex-press device could not be blocked by silicone oil and no oil droplets could be leaked into the surgical bleb, allowing for a competent filtration. In our first case, even if both surgical procedures were effective at reducing ocular hypertension, the IOP was 3.6 mmHg higher in the trabeculectomy group (11.1 ± 10.8 months, range 3.9-39.3) than in the Ex-PRESS group (18.8 ± 14.3 months, range 5.5-43.8). This could be related to an anomalous fibrotic proliferation of the bleb induced by higher stress during the surgical procedures on the anterior chamber during trabeculectomy.

Any surgical approach on vitrectomized eyes has to preserve the anterior and posterior chambers in order to avoid hypotony and its complications of retinal detachment, choroidal hemorrhage, and choroidal effusion (26, 27). Some authors showed that there was a lower incidence of hypotony with the Ex-PRESS implant compared to trabeculectomy (17, 28). We did not record any complications with either technique, but we prefer the Ex-PRESS technique because of the lower incidence of hypotony, which is due to the reduced flow rate through the 50 lumen of the shunt (29). Developments in ophthalmic surgery have been focused on smaller incisions. The Ahmed glaucoma valve could be recommended to manage silicone oil ocular hypertension (30), but mini invasive techniques, such as the EX-PRESS shunt, seem more reliable. The present report is the first one-year long comparison of the efficacy of a trabeculectomy compared to the Ex-PRESS® P50 glaucoma filtration device, based on a retrospective analysis of patients with ocular hypertension secondary to pars plana vitrectomy and silicone oil tamponade. We found that the Ex-PRESS implant could properly manage ocular hypertension induced by silicone oil tamponade and improved patient outcomes and visual recovery.
